# Evolution of the Insect Desaturase Gene Family with an Emphasis on Social Hymenoptera

**DOI:** 10.1093/molbev/msu315

**Published:** 2014-11-24

**Authors:** Martin Helmkampf, Elizabeth Cash, Jürgen Gadau

**Affiliations:** ^1^School of Life Sciences, Arizona State University

**Keywords:** desaturase genes, gene duplication, social Hymenoptera, chemical communication

## Abstract

Desaturase genes are essential for biological processes, including lipid metabolism, cell signaling, and membrane fluidity regulation. Insect desaturases are particularly interesting for their role in chemical communication, and potential contribution to speciation, symbioses, and sociality. Here, we describe the acyl-CoA desaturase gene families of 15 insects, with a focus on social Hymenoptera. Phylogenetic reconstruction revealed that the insect desaturases represent an ancient gene family characterized by eight subfamilies that differ strongly in their degree of conservation and frequency of gene gain and loss. Analyses of genomic organization showed that five of these subfamilies are represented in a highly microsyntenic region conserved across holometabolous insect taxa, indicating an ancestral expansion during early insect evolution. In three subfamilies, ants exhibit particularly large expansions of genes. Despite these expansions, however, selection analyses showed that desaturase genes in all insect lineages are predominantly undergoing strong purifying selection. Finally, for three expanded subfamilies, we show that ants exhibit variation in gene expression between species, and more importantly, between sexes and castes within species. This suggests functional differentiation of these genes and a role in the regulation of reproductive division of labor in ants. The dynamic pattern of gene gain and loss of acyl-CoA desaturases in ants may reflect changes in response to ecological diversification and an increased demand for chemical signal variability. This may provide an example of how gene family expansions can contribute to lineage-specific adaptations through structural and regulatory changes acting in concert to produce new adaptive phenotypes.

## Introduction

Gene families are sets of homologous genes generated by gene duplication events that often display functional similarity and frequently change in size along phylogenetic lineages (Tatusov et al. 1997). Expansion of gene families may occur due to gene duplication and subsequent divergence, whereas gene loss due to deletion or pseudogenization may lead to gene family contraction. The resulting turnover of genes has presumably been one of the driving forces behind the phenotypic differentiation between species (Olson 1999; Lynch and Conery 2000; Ranson et al. 2002; Robertson et al. 2003; Hahn et al. 2007). Both stochastic processes as well as natural selection influence the size of gene families (Hahn et al. 2005). Although there is dispute in the literature about the relative importance of structural versus regulatory changes (Hoekstra and Coyne 2007; Stern and Orgogozo 2008), both are likely important and may even act simultaneously, for example, a recently duplicated gene can acquire a novel function, but at the same time the expression of this novel gene can vary significantly between species or castes (see below). Gene families that are involved in the perception or production of large varieties of semiochemicals (e.g., olfactory and gustatory receptors) or detoxification (e.g., cytochrome P450 monooxygenases and glutathione-S-transferases) tend to gain and lose genes rapidly, which seems to be correlated with changes in life history and ecology (Robertson et al. 2003; Emes et al. 2004; Feyereisen 2006; Després et al. 2007; Guo and Kim 2007; McBride and Arguello 2007; Gardiner et al. 2008; Hansson and Stensmyr 2011).

In insects, chemical communication is a key attribute of recognition and communication, and a huge diversity of chemicals involved in these processes have been described (Greenfield 2002; Blomquist and Vogt 2003; Schulz 2004; Matthews RW and Matthews JR 2010). Semiochemicals help mediate interactions between organisms, and can be species-specific, sex-specific, and, in the case of eusocial insects, colony-, caste-, and task-specific. Insect recognition systems use semiochemicals that function both intraspecifically and interspecifically, and are categorized according to the species-relationship, behavior, and contextual environment in which they are emitted and received. Understanding the roles, developmental pathways, and evolution of insect chemical communication systems has been an exciting challenge to biologists for over five decades. Many initial studies of insect semiochemicals were focused on identifying the components of pheromones and how different compounds affected physiology and behavior. The first of these, bombykol, was identified as an important sex pheromone in the silkworm moth, *Bombyx mori* (Butenandt et al. 1959). Later, when its complete metabolic pathway was described, the desaturase gene *Bmpgdesat1* was found to be critically involved in both the production and perception of bombykol (Moto et al. 2004). In the years since, thousands of other insects and their semiochemicals have been investigated (catalogued in an online database, the Pherobase, http://www.pherobase.com, last accessed November 21, 2014), which have raised many new questions regarding their contribution to complex evolutionary processes including speciation, symbiosis, and sociality. With the ongoing advancement of chemical analysis and synthesis techniques, and the relatively recent advent of genomic and transcriptomic tools, new avenues of insight are now possible for our comprehension of these complex communication systems in insects. Despite these advancements, relatively few genes involved in the synthesis of species-specific semiochemicals are actually known (Dallerac et al. 2000; Labeur et al. 2002; Roelofs et al. 2002; Moto et al. 2004; Chertemps et al. 2006; Niehuis et al. 2013).

The chemical components of insect communication systems have been studied extensively in Coleoptera, Diptera, Lepidoptera, and social Hymenoptera (Symonds and Elgar 2008). A majority of the compounds identified have been acetates, alcohols, aldehydes, ketones, and long-chain hydrocarbons (10–30C), many of which contain carbon–carbon double bonds in varied positions and spatial formations (El-Sayed 2014). Since the 1980s, numerous studies have been conducted with the goal of understanding how insect semiochemicals are biosynthesized, that is, de novo or from minimally modified, environmentally obtained chemical precursors, and furthermore, how specific modifications like hydrocarbon chain length alteration and carbon–carbon double bond introduction are made (Tillman et al. 1999). In the cockroach *Blattella germanica*, for example, the female contact sex pheromone—a dimethyl ketone hydrocarbon—is synthesized de novo through a series of steps following a fatty acid synthesis pathway (Chase et al. 1992). Similarly, in several species of dipterans and lepidopterans, a variety of semiochemicals are synthesized from fatty acid precursors such as myristic acid, palmitic acid, and oleic acid, which are then modified through subsequent biosynthetic steps (Bjostad and Roelofs 1981, 1983; Dillwith et al. 1981; Wicker-Thomas et al. 1997; Dallerac et al. 2000). Among the insects studied to date, a wide variety of semiochemical biosynthetic modification steps have been identified, for example, acetylation, aromatization, decarboxylation, desaturation, elongation, hydrolysis, hydroxylation, methyl-branch incorporation, oxidation, and reduction reactions (Tillman et al. 1999). Although all of these steps are likely important for semiochemical biosynthesis generally, desaturation appears to be especially important for the structural variation in semiochemicals (Knipple et al. 2002; Roelofs and Rooney 2003; Fang et al. 2009). This is due to the fact that desaturation occurs on a diverse range of substrates with both *cis* (Z) and *trans* (E) stereoselectivities, which gives rise to unsaturated compounds with variation in chain length, double-bond number, double-bond position, and double-bond orientation. Because of this, a large number of insect semiochemical studies in Lepidoptera, and a moderate number in Diptera, have focused on understanding the biochemical and genetic diversity of desaturation reactions in recent years (Knipple et al. 2002; Roelofs and Rooney 2003; Hashimoto et al. 2008; Fang et al. 2009; Keays et al. 2011).

The primary group of proteins responsible for desaturation reactions are the desaturases, which are specialized to introduce carbon–carbon double bonds into fatty acyl chains, and are categorized into two main phylogenetic groups (Los and Murata 1998; Sperling et al. 2003). One of these two groups includes the soluble acyl-acyl carrier protein (ACP) desaturases, which are largely found in the plastids of higher plants (Sperling et al. 2003), and are involved in the conversion of saturated fatty acids to monounsaturated fatty acids, for example, oleic acid synthesis (Kachroo et al. 2007). The second group is made up of membrane-bound acyl-lipid desaturases and membrane-bound acyl-coenzyme A (CoA) desaturases. Evidence suggests that a subset of these proteins may be distantly related to the acyl-ACP desaturases (Shanklin and Cahoon 1998; Sperling et al. 2003). Acyl-lipid desaturases appear to be primarily limited to plants and cyanobacteria (Los and Murata 1998), whereas acyl-CoA desaturases are more ubiquitously found in animals, yeast, and fungi, as well as many bacteria (Sperling et al. 2003). These membrane-bound desaturases are important for basic biological processes, including lipid metabolism, cell signaling, and maintaining fluidity in lipid membranes in response to changing temperature (Hazel and Williams 1990; Vigh et al. 1993; Tiku et al. 1996; Pyne S and Pyne N 2000; Miyazaki and Ntambi 2003). What is more, the biochemical functions of membrane-bound desaturases are quite diverse, in that they have been identified to catalyze at least 12 different regioselectivities (i.e., Δ4 − Δ15). However, regioselective function has been shown to inconsistently match with overall sequence similarity, which has made desaturase genes historically challenging to categorize (Ternes et al. 2002; Sperling et al. 2003; Tripodi et al. 2006).

In the past three decades, the functional characterization of desaturase genes has been carried out in a wide variety of organismal models. A key function is their role in the biosynthesis of mono- and poly-unsaturated fatty acids in a range of organisms, including cyanobacteria (Murata and Wada 1995), protists (Tripodi et al. 2006), fungi (Stukey et al. 1989), plants (Domergue et al. 2005; Smith et al. 2013), nematodes, (Watts and Browse 2000; Zhou et al. 2011), insects (Zhou et al. 2008), and mammals (de Antueno et al. 2001; Miyazaki et al. 2001). Furthermore, a number of acyl-CoA desaturase genes have been demonstrated to be crucially involved in semiochemical biosynthesis in many solitary insects, for example, *Drosophila* fruit flies (Dallerac et al. 2000; Fang et al. 2002; Labeur et al. 2002), the silkworm *B. mori* (Moto et al. 2004), and several additional lepidopteran species (Knipple et al. 2002). With this growing body of functional data, Hashimoto et al. (2008) found that membrane-bound desaturase genes could be subdivided into four subfamilies: 1) First Desaturases (primarily Δ9 and Δ11 desaturases), which introduce the first double bond into the saturated acyl chain; 2) Omega Desaturases (Δ12 and Δ15 desaturases), which introduce a double bond between an existing double bond and the acyl end; 3) Front-End Desaturases (Δ4, Δ5, and Δ6 desaturases), which introduce a double bond between an existing double bond and the carboxyl end of an acyl chain; and 4) Sphingolipid Desaturases (sphingolipid Δ4 desaturases), which introduce a double bond into sphingolipids at the Δ4 position. In this study, we adopted Hashimoto’s nomenclature.

Given the role of acyl-CoA desaturases in insect semiochemical production, these genes have recently become an interesting family for study in social insects (Smith CD, et al. 2011; Smith CR, et al. 2011; Suen et al. 2011; Simola et al. 2013), and offer a promising pathway to further our understanding of their recognition systems (Tsutsui 2013). In this study, we determined the diversity of acyl-CoA desaturase genes in 15 insects emphasizing the variation in social Hymenoptera (ants and bees). These desaturases have been demonstrated to be involved in the production of alkenes as part of the cuticular hydrocarbon profile of *Drosophila* (Dallerac et al. 2000; Fang et al. 2002; Labeur et al. 2002), and are crucial for courtship behavior in this genus (Chertemps et al. 2006; Bousquet et al. 2012). Furthermore, evidence suggests that changes in gene number and expression of desaturases affect semiochemical diversity between closely related insect species (Takahashi et al. 2001; Knipple et al. 2002; Roelofs and Rooney 2003; Greenberg et al. 2006; Xue et al. 2007; Fang et al. 2009). As social insects have a highly developed communication system, largely based on cuticular hydrocarbons (Hölldobler and Wilson 1990; Blomquist and Bagnères 2010), we expected an expansion of the desaturase gene family in social Hymenoptera. The annotation of the complete acyl-CoA desaturase repertoire allowed us to study the evolutionary history and mechanisms generating novel genes in these lineages. Furthermore, we present sex- and caste-specific gene expression patterns of three ant species, highlighting the importance of regulatory in addition to structural changes for the evolution of new phenotypes.

## Results

### Gene Annotation and Nomenclature

Searching the genome assemblies and predicted gene sets of 15 insect species allowed us to identify 218 putatively functional acyl-CoA desaturase genes characterized by a fatty acid desaturase type I domain (table 1). We manually improved upon the annotation of many of these genes, with particular emphasis on the seven ant species represented in this study. Our comprehensive search also revealed 75 putative pseudogenes characterized by an interrupted reading frame, short length (<250 amino acids or two-thirds of the average desaturase gene length in *Drosophila melanogaster*), or the lack of a fatty acid desaturase type I domain. Although some of these genes may result from sequencing or genome assembly artifacts, most are likely remnants of once functional genes. Additionally, some genes may have been missed during annotation, yet their number is likely to be very small because we not only searched the genome assemblies for all 15 species, but also unassembled contigs for 8 of the 15 species (supplementary table S1, Supplementary Material online). A small number of genes could not be equivocally identified as either functional or nonfunctional.

**Table 1.** Number of Putatively Functional and Pseudogenized (in parentheses) Acyl-CoA Desaturase Genes in Insects.

	Desat A1	Desat A2	Desat B	Desat C	Desat D	Desat E	Ifc	Cyt-b5-r	Total
*Acyrthosiphon pisum*	4	2	2	1	0	1	1	2 (1^a^)	13 (1^a^)
*Tribolium castaneum*	2	9 (1)	1	1	1	1	1	2	18 (1)
*Bombyx mori*	8	1	7	2 (1)	1	1	1	2	23 (1)
*Drosophila melanogaster*	3	1	1	0	1	1	1	2	10 (0)
*Anopheles gambiae*	2	1	3 (1)	1 (1)	1	1 (1)	2	2	13 (3)
*Nasonia vitripennis*	2	1	8	4	0	1	1	2	19 (2^b^)
*Apis mellifera*	1	4 (1)	1	0	1	2	1	2	12 (1)
*Bombus terrestris*	1	1	1	0	1	1	1	1	7 (1^b^)
*Harpegnathos saltator*	2	2	8	1	1	1	1	1	17 (0)
*Camponotus floridanus*	1	3	6	1	1	1	1	3 (2^a^)	17 (4^a^^,^^b^)
*Linepithema humile*	1 (1)	1 (5^a^)	7 (22)	0	1	1	1	1	13 (28^a^)
*Pogonomyrmex barbatus*	1 (3)	3 (2)	4 (2)	0	1	1	1	1	12 (7)
*Solenopsis invicta*	5 (18)	3	2 (1)	0	1	1	2	2 (3)	16 (22)
*Atta cephalotes*	1	2	6 (2)	0	1	1	1	1	13 (2)
*Acromyrmex echinatior*	1	2	8	0	1	1	1	1	15 (0)
Total	35 (22)	36 (10^a^)	65 (28)	11 (2)	13 (0)	16 (1)	17 (0)	25 (6^a^)	

^a^Includes partially sequenced or otherwise ambiguous genes which may be either functional or pseudogenized.

^b^Includes pseudogenes that could not be assigned unambiguously to a particular desaturase subfamily.

**Fig. 1.**
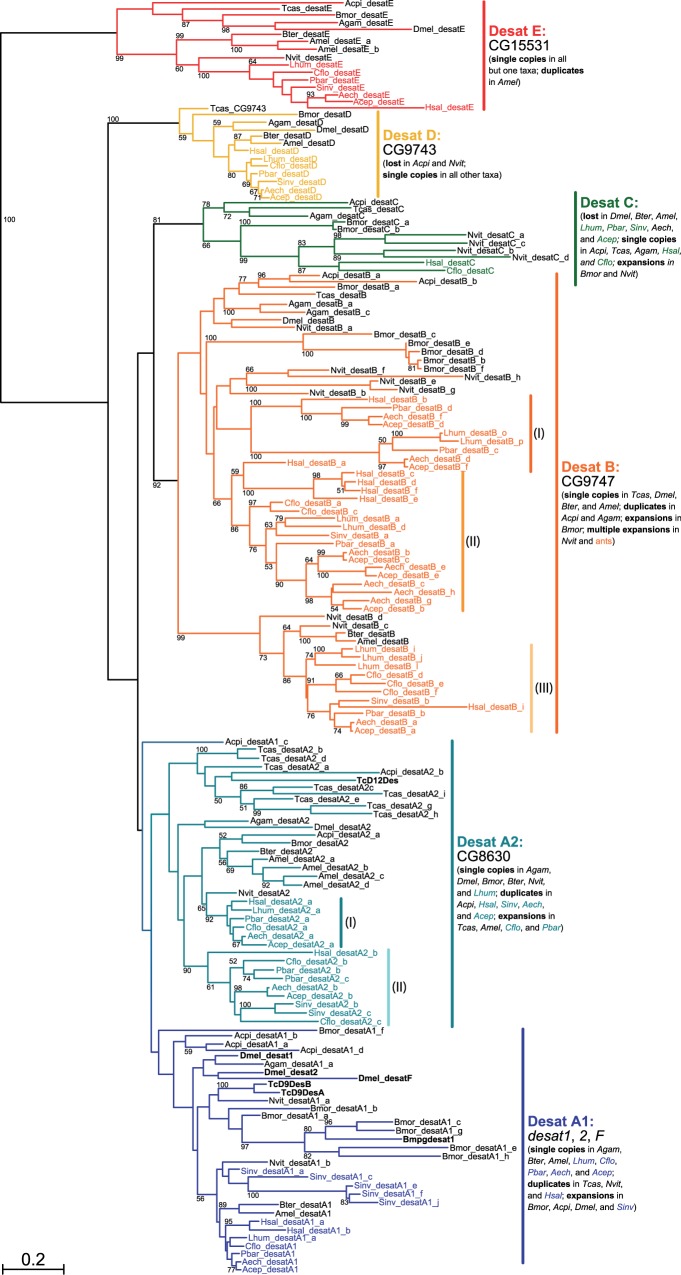
Reconstruction of the phylogeny of insect First Desaturase genes, illustrating the division of the gene family into six subfamilies. The unrooted maximum-likelihood tree was obtained from 170 genes of 15 species, with confidence values at the edges derived from 1,000 rapid bootstrap replicates. Gene names follow the updated nomenclature proposed in this study, except for genes that have previously been characterized in the literature (in bold). Species are indicated by four-letter prefixes as follows: Aech, *Acromyrmex echinatior*; Acep, *Atta cephalotes*; Cflo, *Camponotus floridanus*; Hsal, *Harpegnathos saltator*; Lhum, *Linepithema humile*; Pbar, *Pogonomyrmex barbatus*, Sinv, *Solenopsis invicta* (all ants, in color); Acpi, *Acyrthosiphon pisum*; Amel, *Apis mellifera*; Agam, *Anopheles gambiae*; Bmor, *Bombyx mori*; Bter, *Bombus terrestris*; Dmel, *Drosophila melanogaster*; Nvit, *Nasonia vitripennis*; and Tcas, *Tribolium castaneum*.

According to our similarity-based homology assessment and phylogenetic analyses (fig. 1 and supplementary figs. S1 and S2, Supplementary Material online), insect acyl-CoA desaturases comprise eight orthologous groups. These subfamilies are referred to as Desat A1 (*desat1*, *2*, *F*), A2, B, C, D, E, Ifc (*ifc* [*infertile crescent*]), and Cyt-b5-r (*Cyt-b5-r* [*Cytochrome b5-related*]) in this study (designated *D. melanogaster* genes in parentheses). Genes of subfamilies Desat A1 through E have previously been described as First Desaturases, predominantly stearoyl-CoA Δ9 and Δ11 desaturases that introduce the first double bond at the 9th or 11th position of a saturated acyl chain (Hashimoto et al. 2008). In contrast, Ifc genes are putatively Sphingolipid Desaturases (Hashimoto et al. 2008) with Δ4 activity (Ternes et al. 2002), and bear little sequence similarity to the remaining desaturase genes. The same is true for Cyt-b5-r genes, whose molecular function is unknown. The latter two groups were therefore treated separately in the phylogenetic analyses.

All subfamilies form highly supported monophyletic groups, with the exception of Desat A1 and A2, whose monophyly and sister-group relationship is only weakly supported in the main analysis (fig. 1). However, this relationship is corroborated by the taxonomic distribution of these genes, indicating a deep split during insect evolution, as well as the increase of confidence values for each clade after removing a small number of divergent genes. The uncertain but likely sister-group relationship between Desat A1 and A2 is reflected in the names of these subfamilies. A possible sister-group relationship could also be inferred for Desat B and C. Overall, the phylogenetic relationships between the subfamilies could not be resolved confidently, though. Due to a lack of suitable outgroups (high sequence divergence would have led to a loss of ingroup information during sequence alignment and editing), the direction of evolution could also not be determined in the main analysis, leaving the tree unrooted. A pruned data set with additional noninsect eukaryote desaturase genes was also found insufficient to resolve this issue (supplementary fig. S3, Supplementary Material online).

Based on these phylogenetic results, we propose a new nomenclature for insect First Desaturase genes. Following a four-letter abbreviation of the species name (e.g., *Dmel* for *D. melanogaster*), this nomenclature incorporates the subfamily name as outlined above (e.g., *desatE* for subfamily Desat E) and a one-letter designation for each paralog copy. Paralog designation was chosen independently of phylogenetic position within each subfamily and includes putatively functional genes and putative pseudogenes. Well-supported subclades within subfamilies are thus not indicated in gene names, and only illustrated in fig. 1 (e.g., Desat B I, II, and III). We chose not to adopt a naming scheme that fully reflects the homology relations as resolved by the phylogenetic analyses presented here (Van der Heijden et al. 2007) because of the lack of confidence in some parts of the gene tree. Subsequent phylogenetic analyses with improved resolution and accuracy might render such names obsolete. We also suggest retaining established gene names for functionally annotated genes of model organisms (e.g., *D. melanogaster desat1*, *2*, and *F*) to avoid confusion with already published results.

### Gene Copy Variation

The insect acyl-CoA desaturase subfamilies differ considerably in the number of genes and the complexity of their evolutionary history. With an average number of genes per insect species of 0.9 and 1.1, subfamilies Desat D and E are mostly comprised single-copy genes, although rare cases of lineage-specific gene duplication and loss can be observed as well. Desat C also represents a small, yet slightly more complex group. Besides limited expansions in *B. mori* and *Nasonia vitripennis*, multiple independent cases of gene loss can be inferred from the gene tree, which results in an average number of genes across insects of just 0.7. Multiple losses have particularly affected Hymenoptera, in which only the ants *Harpegnathos saltator* and *Camponotus floridanus* have retained a functional copy apart from *N. vitripennis*. Desat C is also the only one lacking a copy in *D. melanogaster*, exposing the risk of overly relying on homology assessment by the reciprocal BLAST method against a single model organism. All three subfamilies mentioned above have in common that evidence for once functional genes is missing in almost all insects, the only exception being two putative pseudogenes identified in *Anopheles gambiae*. This suggests that the observed gene loss has occurred sufficiently in the past to eradicate all traces of former genes, and that little gene turnover (duplication followed by loss) has taken place more recently.

Similarly, the subfamilies Ifc and Cyt-b5-r also lean toward low copy numbers. However, although Ifc is almost exclusively composed of single-copy genes, most insect species studied here possess two Cyt-b5-r copies. Interestingly, these duplications have occurred independently in all species (supplementary fig. S2, Supplementary Material online).

In contrast, subfamilies Desat A1, A2, and B are characterized by a much higher number of genes and a more dynamic evolutionary history involving frequent episodes of gene gain and loss in multiple lineages. With 2.3 genes on average across insects, subfamily A1 features mostly single-copy genes and duplicates next to several significant lineage-specific expansions in *Acyrthosiphon pisum*, *D. melanogaster* (*desat1*, *desat2*, and *desatF*), *Solenopsis invicta*, and most notably *B. mori* (eight genes). Ants are the only taxon in which we were able to find putative pseudogenes, including 18 in *S. invicta*, suggesting a high rate of gene turnover in this lineage. Similarly, 2.4 genes on average were found in subfamily A2 across insects. Large expansions here include *Apis mellifera* and most notably *Tribolium castaneum* (nine genes). The gene tree topology also suggests that the gene copies in ants can be traced back to one of two ancestral genes that originated in the Hymenopteran lineage before the divergence of ants (fig. 1).

Finally, Desat B forms the single largest group of desaturase genes in insects with 4.3 genes per species on average. Apart from a substantial, recent expansion in *B. mori* (seven genes), the vast majority of these genes are found in Hymenoptera, where multiple episodes of gene family expansion and contraction can be inferred from the phylogenetic tree. The first of these episodes seems to have occurred near or after the evolutionary origin of Hymenoptera, but before the emergence of ants, giving rise to three subclades (I, II, and III) represented in all ant and some of the nonant hymenopteran taxa (some genes have presumably been lost in *N. vitripennis* and the two bee species). This initial expansion was followed by more recent, lineage-specific expansions in most ant species and, to a lesser degree, *N. vitripennis*. Further evidence for considerable gene turnover regarding this subfamily in ants comes from a number of putative pseudogenes, all except one of which are found in ants. The highest number was observed in *Linepithema humile*, whose genome harbors no less than 22 pseudogenes, more than any other lineage with regard to desaturases.

### Genomic Organization in Insects

**Fig. 2.**
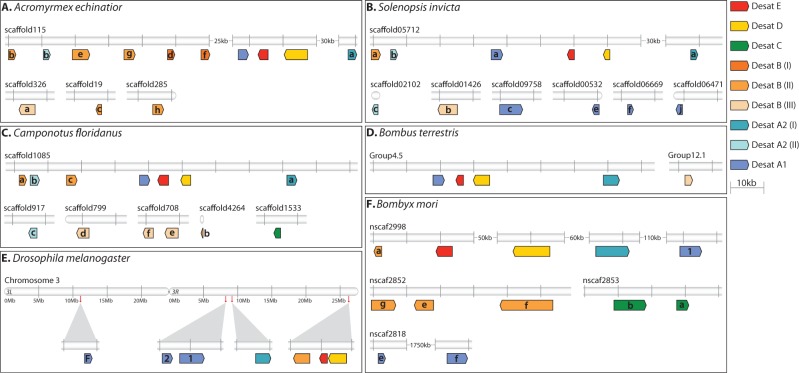
Genomic organization of First Desaturase genes in insects: *Acromyrmex echinatior* (*A*), *Solenopsis invicta* (*B*), *Camponotus floridanus* (*C*), *Bombus terrestris* (*D*), *Drosophila melanogaster* (*E*), and *Bombyx mori* (*F*). Scaffold names refer to the genome assemblies listed in supplementary table S1, Supplementary Material online. Closed scaffold symbols indicate scaffold ends, whereas open symbols indicate that the illustrated region is located away from scaffold ends. Colors and lower case letters are used to identify genes as presented in the phylogeny (fig. 1).

We studied the location, order, and orientation of desaturase genes to shed light on the mechanisms that generated new genes and the evolutionary history of the gene family in insects. In five of seven ants, the majority of functional desaturase genes are located on the same scaffold spanning a region of 100–150 kb, and are highly conserved with respect to order and orientation (fig. 2*A–C*). In *H. saltator* this cluster is broken up into two scaffolds, but the position of the genes on these scaffolds suggests that they still map to the same chromosomal region. Only in *L. humile* most genes are found on separate scaffolds, a fact that might be partially explicable by the low degree of contiguity of the assembly. A notable exception to the microsynteny exhibited by most desaturase genes in ants is made by members of the Hymenoptera-specific Desat B subclade I (see fig. 1), which are consistently found on a different scaffold. The position of genes on the scaffolds and their sizes suggest that these genes and the desaturase core cluster are effectively unlinked, and most likely situated in different chromosomal regions. Many additional members of the highly expanded subfamilies Desat B and Desat A, both functional and pseudogenized, are located on their own scaffolds as well. Some form smaller, often tandemly arrayed clusters like Desat B genes in *L. humile* and *H. saltator*, whereas others are dispersed across many private scaffolds like Desat A1 genes in *S. invicta*. Finally, another exception is made by genes of the subfamily Desat C, which have only been retained by *H. saltator* and *C. floridanus*, and the ant orthologs of *ifc* and *Cyt-b5-r*, all of which are also found on separate scaffolds.

In the remaining Hymenoptera, the arrangement is similar (fig. 2*D*). In both bee species, the desaturase core cluster spans about 80 kb (split across two scaffolds in *Ap. mellifera*, with additional Desat A2 genes occupying another). However, the single Desat B copy in bees is situated on separate scaffold at least megabases away. The situation is slightly more complicated in the wasp *N. vitripennis*, where the core cluster is broken up into two groups located on different, unlinked scaffolds. As in ants, several Desat B paralogs are found in close proximity to the *desat1* ortholog, whereas others reside on their own scaffolds. Likewise, ifc and Cyt-b5-r genes occupy a different genomic region than all other desaturase genes.

Dipteran First Desaturase genes are found on a single chromosome arm—3R in *D. melanogaster* (except *desatF*, which is on 3L) and 2R in *A. gambiae*—which displays a high degree of synteny between the species (Zdobnov et al. 2002). In both species, a tightly linked block of *CG9747*, *CG15331*, and *CG9743* or their orthologs is separated from *desat1* and *CG8630* by several to many megabases (only the relative position of *CG8630* differs between both species) (fig. 2*E*).

This synteny is also displayed by *B. mori*, the closest relative of the Dipteran species in our taxon sampling. Here, members of all First Desaturase subfamilies are found in a 125-kb region, in an order reminiscent of that in *A. gambiae* (fig. 2*F*). As seen in ants, genes produced by recent, lineage-specific expansions can be traced back to different genomic regions, and sometimes form smaller clusters (e.g., several Desat B paralogs). *Tribolium castaneum* core genes occupy two smaller, unplaced scaffolds and may therefore be microsyntenic as well, whereas more recently generated Desat A2 paralogs comprise several independent clusters. In contrast to all other species studied, only *Ac. pisum* provides little evidence for synteny. With the exception of three genes from two subfamilies, Desat A1 and Desat E, all genes are scattered across different scaffolds. Like in Hymenoptera, ifc and Cyt-b5-r genes are physically unlinked from First Desaturase genes in all non-hymenopteran species studied.

### Selective Forces Acting on Desaturase Genes

The results of the signature of selection analysis provide little evidence for positive selection acting on desaturase genes in insects (table 2). Under the most basic model M0, which assumes that the ratio of nonsynonymous to synonymous substitutions is invariable among sites and branches, *ω* (=*d*_N_/*d*_S_) ranges from 0.08 to 0.21 among the desaturase subfamilies. More realistically allowing *ω* to vary among sites following a beta-distribution (models M7 and M8) results in a strongly L-shaped distribution in all subfamilies, indicating that most sites represent very small *ω* values and are thus under purifying selection. Adding another class for sites under positive selection (M8) results in a significantly better fit according to likelihood ratio tests (LRT) in two subfamilies, Desat A2 and Desat D. However, the estimated proportion of positively selected sites is either very small, or the *ω* values for this site class are very close to 1, attesting neutral rather than positive selection. Moreover, Bayes empirical Bayes analyses (Yang et al. 2005) fail to detect sites under positive selection with posterior probability greater than 95% in all subfamilies.

**Table 2.** Signatures of Selection Acting on First Desaturase Genes in Insects.

Model—Parameters	Desat A1 (*desat1*)	Desat A2 (CG8630)	Desat B (CG9747)	Desat C —	Desat D (CG9743)	Desat E (CG15531)
Basic/site models						
M0: *ω*	0.09	0.13	0.21	0.08	0.09	0.14
M7: *p*, *q*	0.65, 5.09	0.51, 2.50	0.62, 1.81	0.78, 5.23	0.33, 2.18	1.15, 6.03
M8: *p*_1_, *ω*	0.00, 3.74	0.02, 1.08	0.00, 1.00	0.01, 1.18	0.06, 1.00	0.01, 1.00
Branch models (ants)						
B0: *ω*_0_ (*ω*_1_ = 1)	0.01	0.09	0.08	0.05	0.04	0.10
BA: *ω*_0_, *ω*_1_	0.05, 0.14	0.10, 0.16	0.11, 0.27	0.06, 0.15	0.05, 0.12	0.11, 0.16
Branch-site models (ants)						
A0: *p*_2a_ (*ω*_2_ = 1)	0.07	0.10	0.08	0.16	0.03	0.12
AA: *p*_2a_, *ω*_2_	0.07, 1.00	0.10, 1.00	0.08, 1.00	0.16, 1.00	0.03, 1.00	0.12, 0.09
LRT, *P*						
M7 versus M8	1	*0.037*	1	0.678	*0.001*	0.698
M0 versus BA	*0.010*	*<0.001*	*0.001*	*<0.001*	*<0.001*	*<0.001*
B0 versus BA	*<0.001*	*<0.001*	*<0.001*	*<0.001*	*<0.001*	*<0.001*
A0 versus AA	1	1	1	1	1	1

Note.—Select parameter estimates and LRT results are shown for each of the six subfamilies in this table. *ω*, ratio of nonsynonymous to synonymous substitution rates; *p*, *q*, beta distribution shape parameters (M7); *p*_1_, proportion of sites under positive selection (M8); *ω*_0_, *ω*_1_, background and foreground *ω* values, respectively; *P*, likelihood ratio test *P* value (significant results in italic).

We then looked whether strongly expanded subclades of desaturase genes, and ant-specific genes in general, evolved under different selective pressures than the remaining (background) genes by assigning different *ω* values to foreground and background branches. Tested foreground branches included all ant-specific genes in each subfamily, as well as the *B. mori*-specific expansions in Desat A1 and Desat B, and the *T. castaneum*-specific expansion in Desat A2. In all cases, the branch-specific model (BA in table 2, shown for ants only) proved to fit the data significantly better than the basic model M0. Foreground *ω* values with respect to ants were significantly higher than background *ω* values in all subfamilies, with the biggest difference found in Desat B (0.27 and 0.11, respectively). In contrast, foreground *ω* values for *B. mori* and *T. castaneum* were significantly smaller than background *ω* values. Fixing *ω* for the foreground branches at 1 (model B0) did not lead to an improved fit over the unconstrained model BA because foreground *ω* values were estimated to be much smaller than 1.

Allowing *ω* to vary both among sites and branches using a branch-site model did not provide evidence for sites under positive selection in ants only (AA in table 2). In fact, *ω* for sites allowing positive selection in ants only was estimated to be 1 in all subfamilies, confirming the role of purifying selection in desaturase genes across insect lineages. The branch-site test of positive selection was therefore nonsignificant.

### Gene Expression in Ants

**Fig. 3.**
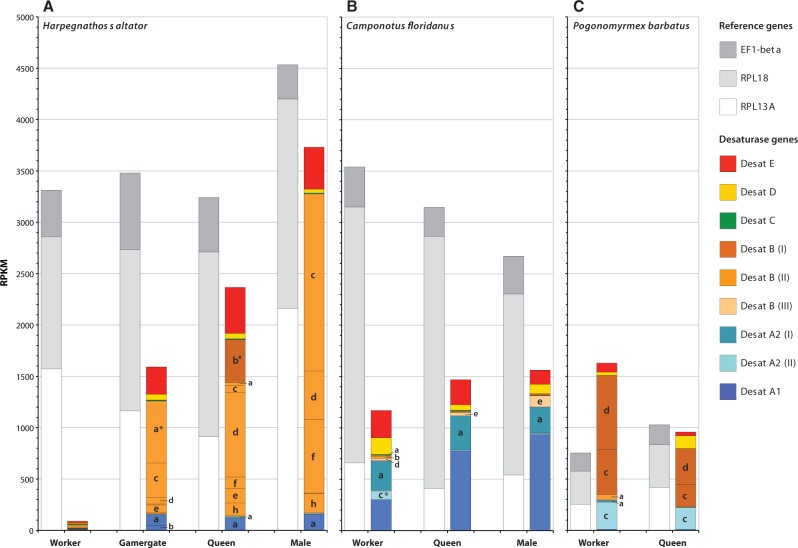
Gene expression levels of reference genes and First Desaturase genes in different sexes and castes of three ant species: *Harpegnathos saltator* (*A*), *Camponotus floridanus* (*B*), and *Pogonomyrmex barbatus* (*C*). Data are shown in RPKM, and were obtained from independent RNA-seq experiments. Medium gray, light gray, and white bars represent reference genes (EF1-beta, RPL18, and RPL13A, respectively), whereas colored bars and lower case letters are used to identify desaturase genes as presented in the phylogeny (fig. 1). Asterisks indicate desaturase genes showing strong differential expression in a particular caste within a species. Also note that *C. floridanus* workers were of the “minor” worker caste, queens of all three species were virgin (unmated) queens, and data were not available for *P. barbatus* males.

We found strong differences in the transcription levels of the First Desaturase genes across the sexes and castes in three ant species (fig. 3). The expression of three reference genes, however, did not vary strongly between sexes and castes within a species (coefficients of variation ≤ 0.38) but varied moderately between species (coefficients of variation ≤ 0.70), for example, *Pogonomyrmex barbatus* showed an overall lower expression level than the other two species. Hence, comparisons of expression patterns within a species reflect quantitative differences whereas interspecific comparisons can only be qualitative, that is, presence or absence of clade-specific expression, or expression ratios between clades.

In *H. saltator*, desaturase genes are generally expressed at very low levels (reads per exon kilobase per million [RPKM] < 100) in workers relative to the reference genes. The expression profiles of adult, virgin queens, and males are however dominated by the Desat E ortholog and several Desat B genes, some of which reach very high levels (RPKM > 1,000) (fig. 3*A*). This stands in contrast to *C. floridanus* (fig. 3*B*), where the cumulative expression levels in minor workers, virgin queens, and males are more balanced, and dominated by genes of Desat A1 and A2, whereas Desat B genes are barely represented. However, there is agreement between the two species in the moderate expression of Desat E and Desat D orthologs. *Pogonomyrmex barbatus* expression profiles (fig. 3*C*) resemble more closely those found in *H. saltator* in the fact that Desat B genes are more highly expressed than genes of any other subfamily. However, these genes hail from a different subclade within Desat B (I) than most that are overrepresented in *H. saltator* (II). Also in contrast, *P. barbatus* workers express First Desaturase genes more than virgin queens (data for adult males is not available). Overall expression levels, though similar or lower than in the other two species in absolute terms, seem to be higher relative to the reference genes. Consistent across all three species and most castes seems to be the proportionately moderate expression of the Desat E and Desat D orthologs. Finally, several desaturase genes are strongly overrepresented in particular castes or sexes within a species. Most notably, *desatB_b* in queens and *desatB_a* in gamergates of *H. saltator*, and *desatA2_c* in *C. floridanus* workers were found to be expressed almost exclusively in these castes.

## Discussion

### Evolution of Genomic Organization

Studying the location, order and orientation of the acyl-CoA desaturase genes at the genomic level revealed a high degree of microsynteny within genomes, and strong organizational conservation across insect species. We found a cluster of genes including members of all First Desaturase subfamilies except Desat C in a wide range of species from ants and bees to *B. mori*, with only slight variations in gene order and orientation (fig. 2). Although in *D. melanogaster* and *N. vitripennis*, this cluster has apparently been broken up into smaller blocks by large-scale genomic rearrangements, it is still evident in microsynteny retained within those blocks. Conservation of the cluster-like organization of First Desaturase genes between species separated by millions of years of evolution suggests that these genes are ancestral to holometabolous insects. Hence, homologous recombination by unequal crossing-over seems to be the dominant mechanism generating these genes from a single ancestral gene. As the aphid *Ac. pisum* possesses a full complement of acyl-CoA desaturase genes, these events must have preceded the split between hemipterans and holometabolous insects, even though genomic rearrangements have obliterated most traces of ancient microsynteny in this species. Phylogenetic analyses further suggest that the ancestral expansion of the First Desaturase genes occurred presumably along the insect lineage, as orthologs found in noninsect arthropods, nematodes and vertebrates seem to be derived from duplication events succeeding the divergence between these lineages and insects (Hashimoto et al. 2008; supplementary fig. S3, Supplementary Material online).

The genes making up this ancestral cluster are thus the source from which new, lineage-specific desaturase genes were generated in insects. The genomic organization of these novel lineage-specific genes suggests two mechanisms shaping the evolution of desaturase genes in insects, homologous recombination and chromosomal mutations. Closely linked or even tandemly arrayed gene copies provide evidence that homologous recombination gave rise to many novel genes, both within and outside of the original cluster. For instance, the expansion of Desat B genes in the Attines, *Atta cephalotes* and *Acromyrmex echinatior*, can be traced back to such events (fig. 2*A*). The duplications of the ancestral genes which generated the ant-specific subclades Desat B II and III, and Desat A2 I and II (fig. 1) seem to have involved homologous recombination as well, because extant copies are found in close proximity to each other in most ant species (fig. 2*A–C*). Finally, most recent, species-specific expansions have resulted in tandem arrays of novel genes, for example, the expansion of Desat B subclade II genes in *H. saltator*, Desat A2 genes in *T. castaneum*, and most likely Desat B genes in *B. mori* (fig. 2*F*).

However, the presence of unlinked desaturase genes in many insect species, most notably the members of the ant-specific Desat B subclade I, indicates another mechanism generating novel genes. As functional genes of this category closely resemble gene copies located in the core cluster in terms of intron–exon structure and thus contain intronic sequence, retrotransposition seems not to be involved in the process (Long 2001). Instead, chromosomal mutations like segmental duplication followed by chromosome rearrangement could be responsible, an ill-understood process frequently observed in other animal lineages (Samonte and Eichler 2002). Segmental duplication results in longer stretches of identical sequence, including noncoding intergenic DNA. We did not find evidence for high sequence similarity between regions adjacent to genes located in the core cluster and unlinked genes, although as little selection pressure is expected to act on intergenic DNA, mutations accumulated over time might have erased these traces.

### Evolution of Gene Repertoire

The acyl-CoA desaturase gene family is characterized by a highly dynamic evolutionary history in insects. Careful annotation and phylogenetic analysis of both functional and pseudogenized genes allowed us to infer eight subfamilies which differ strongly in their degree of conservation and frequency of gene gain and loss (fig. 1 and supplementary figs. S1 and S2, Supplementary Material online). Three of the eight subfamilies are characterized by frequent expansions and higher rates of gene turnover, which also do not affect insect lineages equally. Although the First Desaturase subfamilies Desat C, D, and E as well as the Ifc and Cyt-b5-r subfamilies are largely comprised of single-copy genes, subfamilies Desat A1, A2, and B harbor a much higher number of genes (fig. 1). Some cases of gene family expansion, most notably the episode of gene gain that gave rise to the ant-specific Desat B subclades I, II, and III, can be traced back to deeper splits in the insect phylogeny. However, the vast majority of expansions seem to have occurred more recently. Taxa that are disproportionally affected by such lineage-specific expansions include *B. mori*, *T. castaneum*, *N. vitripennis* and most ant species, particularly *H. saltator*, *L. humile*, *C. floridanus*, and *S. invicta* (table 1). Similarly, the majority of pseudogenes are found in only a few species, most notably the two invasive ant species *L. humile* and *S. invicta*, indicating a particularly high rate of gene turnover in these lineages. In contrast, dipteran (*D. melanogaster* and *A. gambiae*) and bee (*Ap*. *mellifera* and *Bombus terrestris*) genomes contain a lower number of functional and nonfunctional desaturase genes on average than most other insects, and do not show extensive lineage-specific expansions (table 1). It is worth noting that the unequal taxonomic distribution of the lineages represented here may mask more ancient expansions in some cases, though. For instance, the expansion of Desat A1 in *B. mori* might have occurred early during Lepidoptera evolution, in line with the long branches characterizing this group of genes in our study (fig. 1). This is further supported by the finding of Knipple et al. (2002), which found orthologous copies of these genes in multiple lepidopteran species. As genome data of more species are becoming available to fill taxonomic gaps, the pattern of gene gain and loss in other lineages might turn out to more closely resemble that observed in ants, with multiple independent episodes of expansion and contraction along their evolutionary trajectories.

The consistently low copy-number and lack of pseudogenes in the subfamilies Desat C, D, and E as well as the subfamilies including *ifc* and *Cyt-b5-r* (fig. 1 and supplementary figs. S1 and S2, Supplementary Material online) suggest that these genes are housekeeping genes, possibly serving a basic function in lipid metabolism pathways. Unsaturated fatty acids, the products of the enzymatic activity of desaturases, are essential for many basic cellular functions, including energy storage, cell signaling, and the regulation of membrane fluidity (Hazel and Williams 1990; Vigh et al. 1993; Tiku et al. 1996; Pyne S and Pyne N 2000; Miyazaki and Ntambi 2003). In contrast, high gene turnover rates in the other subfamilies are more indicative of lineage-specific adaptations and potentially play an important role in the phenotypical differentiation between clades (Olson 1999; Lynch and Conery 2000; Ranson et al. 2002; Robertson et al. 2003; Hahn et al. 2007; Khalturin et al. 2008; Colbourne et al. 2011; Voolstra et al. 2011). Although both stochastic processes as well as selection influence the size of gene families, particularly large differences in gene family size between genomes may be attributed to adaptation (Hahn et al. 2005). We have previously shown that size variation of the acyl-CoA desaturase gene family along the insect phylogeny differs significantly from expectation due to neutral mutation and genetic drift (Simola et al. 2013). Such significant differences in gene family size between clades can be indicative of selection.

Gene duplication is thought to free one copy from the selective pressures operating on the ancestral gene; therefore, we expected to find signatures of relaxed selective constraint or positive selection in the genes of the expanded desaturase subfamilies (Ohno 1970). Unexpectedly, the results of our signature of selection analysis provided little evidence for either, and instead revealed generally strong purifying selection acting on all desaturase genes in insects (table 2). The ratio of nonsynonymous to synonymous substitutions *ω* proved to be slightly higher in the expanded subfamilies Desat A2 and B than in the more conserved subfamilies, but still remains very low (*ω* < 0.25). Desat A1 did not display an elevated ratio *ω*, despite harboring expansions in multiple lineages. Results from the more accurate site model analyses (Yang et al. 2000) revealed that the low average values of *ω* are due to an abundance of sites under purifying selection in contrast to only a small number of sites under neutral or weak positive selection. As even recent gene duplicates from the same species differ by at least 10% on the amino acid sequence level, the low ratios of nonsynonymous to synonymous substitutions cannot be attributed to a lack of sequence variation. We also found no indication of strong differences in the selective pressure acting on lineages defined by large gene expansions in individual subfamilies. Although we were able to detect significantly higher values of *ω* in ant genes in comparison to genes from other taxa, the differences were small and remained indicative of strong purifying selection. The slight relaxation of selective pressure relative to other insects seems more likely to be intrinsic to ants than related to differences in gene turnover and gene family size because both more conserved (Desat C, D, and E) and more variable (Desat A1, A2, and B) subfamilies were affected equally. A reason for this might be the decreased efficiency of selection in ants due to the smaller effective population sizes typical for social insects (Crozier and Pamilo 1996; Gadau et al. 2012). The lack of positive selection in the single-copy desaturase subfamilies is in line with the hypothesis that these genes constitute housekeeping genes performing essential metabolic functions. The strength of purifying selection remains puzzling in the larger subfamilies, though, where redundant copies are expected to evolve more freely and acquire novel, lineage-specific functions. However, it is consistent with small *ω* ratios reported previously from desaturases in *D. melanogaster* (Keays et al. 2011) and *B. mori* (Knipple et al. 2002). These studies included *desat1* and *Bmpgdesat1*, two genes that have been shown to be involved in pheromone production in these two species, and members of the expanded subfamily Desat A1. This observation may indicate that although duplicated desaturase genes can change function rapidly it may not be due to fundamental changes in the enzymatic function and coding sequence of the gene, which would remain under strong purifying selection, but rather in the differential expression of these new copies in time and space.

### Functional Differentiation in Ants

The comparison of the First Desaturase gene expression levels in three ant species provided evidence for both highly conserved as well as species-, sex-, and caste-specific expression patterns depending on desaturase subfamily (fig. 3). Genes from subfamilies Desat D and E are expressed consistently across all categories, supporting the notion that they represent housekeeping genes involved in basic lipid metabolic processes. Desat C genes, which are present only in *H. saltator* and *C. floridanus*, are barely expressed in any category. This may indicate that these genes, which have been lost in many insect species including the bee and most ant species represented in this study, no longer fulfill an essential function in most insects.

In contrast to the single-copy genes mentioned above, genes of the expanded subfamilies Desat A1, A2 and B display variation in gene expression between species, and between sexes and castes within species. Notably, we see no consistent sex- and caste-specific use of orthologous genes between ant species as has been observed for other genes expanded in ants such as *vitellogenin* (Corona et al. 2013). Instead, the expression pattern in each species is largely dominated by either Desat A1 and A2 (*C. floridanus*), or Desat B genes (*H. saltator*, *P. barbatus*), but never both. Moreover, only Desat B genes of subclade II are expressed at high cumulative levels in *H. saltator* (with the exception of *desatB_b* of subclade I in virgin queens), whereas *P. barbatus* exhibits predominantly subclade I gene expression. As these data were compiled from several studies, the differences across species observed here might have been influenced by different laboratory conditions and RNA sequencing methodologies. However, *C. floridanus* and *H. saltator*, which exhibit some of the most pronounced gene expression differences discussed above, were raised in the same laboratory space at Arizona State University under highly similar conditions, and their RNA was sequenced at the same time by the same research group (Bonasio et al. 2010). Additionally, the expression levels of the reference genes within each species show only moderate differences (coefficients of variation ≤ 0.38), whereas the differences between species are somewhat more pronounced (coefficients of variation ≤ 0.70), thus preventing detailed quantitative comparisons. Notwithstanding, we can still compare expression patterns between species in terms of differences in the relative frequency of individual clades. For example, one notable pattern includes the Desat A1 subfamily, which is relatively frequent in *C. floridanus* and *H. saltator*, but almost undetectable in *P. barbatus* (fig. 3). Another, even more convincing example is the expression differences in Desat B subclade I and Desat B subclade II between *P. barbatus* and *H. saltator* in all castes and sexes with the exception of *H. saltator* workers which have an overall reduced expression of all desaturase genes (fig. 3). In *P. barbatus*, Desat B subclade I is very prominently expressed whereas Desat B subclade II shows very little expression. In contrast, *H. saltator* shows the opposite pattern, which cannot be explained by generally lower expression in *P. barbatus*. In contrast to the previous two species, *C. floridanus* has very low expression of both Desat B subclades I and II (fig. 3). Hence, the caste- and sex-specific expression pattern between these three species representing three ant subfamilies (diverged ∼ 100 Ma) demonstrates that each lineage has radically modified the expression patterns of First Desaturase subclades.

The intraspecific expression differences between castes and sexes are not as extreme as the interspecific differences; however, there are significant and possibly functionally relevant caste and sex differences (fig. 3). For instance, *desatB_b* is expressed almost exclusively in *H. saltator* virgin queens, but not workers or males, and only very lowly in gamergates (fig. 3). On the other hand, *desatB_a* expression is specific to *H. saltator* gamergates, whereas *desatB_d* is strongly overrepresented in virgin queen and males, which in turn express much more *desatB_f* than gamergates and virgin queens (fig. 3). Another example includes the worker-specific *C. floridanus* gene *desatA2_c*. These cases of genes originating from recent lineage-specific expansions with differential intraspecific expression patterns are suggestive of gene duplication followed by neofunctionalization (Ohno 1970). Another noteworthy observation regarding First Desaturase gene expression in ants is that these genes do not seem to fulfill metabolically essential roles, as workers in *H. saltator* display very low expression levels, particularly of Desat A1, A2, and B. This pattern of functional differentiation among lineages and within species, and the fact that genes stemming from expanded subfamilies do not seem to be essential for survival, fit the expectation of genes involved in the production of semiochemicals, as described for several desaturase genes in *D. melanogaster* (*desat 1*, *2*, *F*; Dallerac et al. 2000; Fang et al. 2002; Labeur et al. 2002) and *B. mori* (*Bmpgdesat1*; Moto et al. 2004).

Changes in the expression and number of acyl-CoA desaturase genes have been shown to affect the diversity of semiochemicals between closely related insect species (Takahashi et al. 2001; Knipple et al. 2002; Roelofs and Rooney 2003; Greenberg et al. 2006; Xue et al. 2007; Fang et al. 2009). In ants, the use of cuticular hydrocarbons in chemical communication is widespread (Hölldobler and Wilson 1990; Blomquist and Bagnères 2010), and unsaturated compounds such as alkenes have been suspected of providing sufficient diversity to act as key discriminatory compounds (Martin and Drijfhout 2009). Indeed, cuticular hydrocarbon profiles differ strongly between species, sexes, castes, and developmental stages in ants, including *H. saltator*, *C. floridanus*, and *P. barbatus* (Wagner et al. 1998; Liebig et al. 2000; Endler et al. 2004). For instance, cuticular hydrocarbon profiles, including unsaturated compounds, have been shown to undergo a shift when individuals transition from nonreproductive to reproductive status in *H. saltator* (Liebig et al. 2000), a change that may be correlated with the caste-specific gene expression pattern described above. Differences in the relative proportions of various alkenes have also been found between workers and queens of *P. barbatus* (Cash EI, unpublished data). As the evolution of castes in ants added another layer of complexity to the chemical communication system employed by insects, its genetic regulation might have been facilitated by novel genes with variable expression patterns. This case may thus provide an example for both structural and regulatory changes acting in concert to produce a new phenotype.

The production of cuticular hydrocarbons is not the only function of desaturase genes in insects. Desaturases have also been shown to be involved in the synthesis of bombykol (Moto et al. 2004) and other unsaturated compounds serving as volatile pheromones (Roelofs and Rooney 2003). Moreover, many components of insect chemical communication systems that have been studied in Coleoptera, Diptera, Lepidoptera, and social Hymenoptera include carbon–carbon double bonds (El-Sayed 2014), and thus presumably also require the activity of desaturases for synthesis. Many more may still await discovery, including a significant portion of the abundance of recruitment and alarm pheromones and other glandular secretions employed by ants, only a fraction of which have been described (Hölldobler and Wilson 1990). Desaturase gene expansions in solitary species such as *B. mori*, *N. vitripennis*, and *T. castaneum* that rival those seen in ants may be involved in the production of such undiscovered compounds.

Furthermore, differences in the repertoire of insect desaturase genes may also be due to differences in diet and climatic conditions requiring changes in lipid metabolic pathways. Again, the large number and frequent turnover of desaturase genes in many insect lineages may reflect their enormous ecological success and diversity. This seems to be especially plausible for beetles (*T. castaneum*) and ants, two groups that have colonized nearly every terrestrial habitat, and are also extremely diverse in terms of their diet. For instance, ants include generalists (e.g., *C. floridanus*) and specialists (e.g., the leaf-cutters), herbivores, detritivores, omnivores (e.g., *C. floridanus*) and predators (e.g., *H. saltator*), and residents of diverse ecosystems ranging from deserts (e.g., *P. barbatus*) to tropical rainforests (e.g., the leaf-cutters), and thus rely on very different diets and are exposed to very different climatic conditions. The same is true for beetles, one of the most speciose, ecologically diverse, and successful insect lineages. Thus, changes in the desaturase gene repertoire may reflect changes in both ecological niches and chemical communication needs which arose during the evolution of various insect lineages. Indeed, novel genes have been shown to underlie lineage-specific adaptations including responses to changing environmental stimuli (Colbourne et al. 2011; Voolstra et al. 2011). Patterns of repeated size changes are also found in other gene families in ants, such as P450 cytochromes and olfactory receptors, possibly for similar reasons (i.e., the need to detoxify or perceive compounds encountered in new environments, respectively; Simola et al. 2013).

Social organization alone may not require an expanded repertoire of desaturase genes, as demonstrated by the fact that the two bee species represented in this study revealed the smallest number of genes. As opposed to bees, however, ants display a much wider range of social complexity in terms of colony size, number of queens and queen–worker differentiation, and possess a more elaborate chemical communication system (Hölldobler and Wilson 1990). Combined with their ecological diversity, this may explain why the number of desaturase genes is so much higher in ants than in bees. The particularly high number of both functional and nonfunctional genes in *L. humile* and *S. invicta* may even be a testimony to these lineages’ ability to quickly adapt to new ecological niches and changes in social organization in the past, features which more recently became instrumental in their success as invasive species.

## Conclusions

Genomic organization and phylogeny testify that acyl-CoA desaturases represent an ancient gene family characterized by multiple episodes of expansion and contraction during evolution of insects. Subfamilies differ strongly in their degree of conservation and frequency of gene gain and loss, which also do not affect insect lineages equally. Ants display particularly large expansions in three First Desaturase subfamilies, in stark contrast to bees. Hence, eusociality itself cannot explain this pattern of disproportionate gene gain. As the number of genes in *N. vitripennis*, a solitary hymenopteran, rivals that of ants in some parts of the desaturase tree, the richness of desaturase genes in ants seems to be a part of the Hymenopteran heritage that has been lost in bees. What are the driving forces promoting and maintaining this rich gene repertoire in ants? Multiple causes appear to provide plausible explanations: Variation in gene expression between ant species, and more importantly, between sexes and castes within species, suggests functional differentiation of these genes and a role in the regulation of reproductive division of labor in ants. As First Desaturase genes found in these subfamilies are involved in the production of mating signals in *D. melanogaster* and *B. mori*, we hypothesize that the homologous genes in ants serve a role in the elaborate chemical communication system of ants. The expansions observed in ant First Desaturase genes may therefore have provided genetic raw material facilitating social evolution in ants as the evolution of castes and social organization added another layer of complexity to this system. On the other hand, ants also vary considerably in life history traits and the environment they live in, leading to very different diets and exposure to different climatic conditions. Desaturases could therefore have contributed to lineage-specific adaptations with regard to these differences, requiring changes in lipid metabolic pathways. Finally, the dynamic evolution of acyl-CoA desaturases may reflect changes in both ecology and chemical communication systems, responding to ecological diversification and an increased demand for chemical signal variability during ant evolution. This may provide an example for how gene family expansions can contribute to lineage-specific adaptations and how structural and regulatory changes act in concert to produce new adaptive phenotypes. Further studies elucidating the molecular function of acyl-CoA desaturases, and members of the expanded subfamilies in particular, are required to discern their significance for the ecology, chemical communication, and social evolution in ants.

## Materials and Methods

### Identification of Insect Desaturase Genes

Genome assemblies and predicted gene sets of 15 insect species, including seven ant species (*Acr. echinatior*, *At. cephalotes*, *C. floridanus*, *H. saltator*, *L. humile*, *P. barbatus*, and *S. invicta*), three nonant Hymenoptera (*Ap. mellifera*, *Bombus terrestris*, and *N. vitripennis*), and representatives of Diptera (*D. melanogaster* and *A. gambiae*), Lepidoptera (*B. mori*), Coleoptera (*T. castaneum*), and nonholometabolous insects (*Ac. pisum*) were obtained from their respective community databases (supplementary table S1, Supplementary Material online).

We chose all ten acyl-CoA desaturase genes characterized by a fatty acid desaturase type I domain in *D. melanogaster* (Keays et al. 2011), namely CG15531, CG9743, CG9747, CG8630, *desat1*, *desat2*, *desatF*, *ifc*, CG17928, and *Cyt-b5-r* as queries to find homologous sequences in the 14 other species. First, predicted gene sets were searched with BLASTP (Altschul et al. 1997) using an *e* value cutoff of 0.0001 to obtain the majority of putatively functional genes as identified by automatic annotation pipelines. In the case of *At. cephalotes*, *L. humile*, and *P. barbatus*, manually annotated desaturase gene repertoires were already available based on previous work (Suen et al. 2011; Smith CD, et al. 2011; Smith CR, et al. 2011). To identify genes and gene fragments not represented in the predicted gene sets, we also searched the genome assemblies of all 15 species, and unassembled contigs for 8 of 15 species (supplementary table S1, Supplementary Material online) with TBLASTN using an *e* value cutoff of 0.001. Genomic regions surrounding hits that were found to be lacking an existing gene model were then subjected to a GeneWise (Birney et al. 2004) analysis to predict the gene structure. The same strategy was used to guide manual editing of existing gene models that did not align well with the *D. melanogaster* query. Information about all genes used in this study, including their genomic location, is compiled in the supplementary material (supplementary table S2, Supplementary Material online). Nucleotide and amino acid sequences are available from the authors upon request.

Genes were categorized as functional if they contained an open reading frame of at least 250 amino acids (approximately two-thirds of the average desaturase gene length in *D. melanogaster*) and a fatty acid desaturase type I domain (IPR005804) according to InterPro (Hunter et al. 2009). Shorter genes or genes lacking this domain were classified as pseudogenes unless the truncation resulted from unresolved or misassembled sequence in the genome assembly. In rare cases, genes could not be assigned to either category, for example if a substantial part of the gene was masked by unresolved sequence. Fragments on very short scaffolds which were identical in sequence to parts of full-length desaturase genes were assumed to be assembly artifacts and excluded from the analyses.

### Phylogenetic Reconstruction

Preliminary phylogenetic analyses revealed that the *D. melanogaster* genes *ifc* and *Cyt-b5-r* bear little sequence similarity to the remaining desaturases and each other. To improve the quality of the alignment, we therefore excluded all *ifc* and *Cyt-b5-r* orthologs, as well as all pseudogenes and some ambiguous genes from the main phylogenetic analysis. The resulting amino acid data set contained 170 genes classified as First Desaturases by Hashimoto et al. (2008) from 15 species, and was aligned with the L-INS-i algorithm implemented in MAFFT version 7 (Katoh et al. 2002; Katoh and Toh 2008). To remove divergent and poorly aligned positions, we used Gblocks version 0.91b (Castresana 2000) on the lowest stringency settings, resulting in a final alignment of 218 amino acid positions. According to the Akaike Information Criterion corrected for small sample size, ProtTest version 2.4 (Abascal et al. 2005) revealed LG with four discrete gamma rate categories (Le and Gascuel 2008; Yang 1996) as the model of molecular evolution with the best fit to the data (models combining gamma rates and a proportion of invariable sites, G+I, were omitted from the analysis following the argument of redundancy put forward by A. Stamatakis, RAxML version 7.0.4 manual, page 20). Based on this model, a maximum-likelihood tree was reconstructed with RAxML version 7.2.6 (Stamatakis 2006), and nodal confidence values obtained with 1,000 rapid bootstrap replicates (Stamatakis et al. 2008).

Phylogenetic trees were computed as outlined above for Ifc (314 amino acid positions derived from 17 putatively functional genes) and Cyt-b5-r (384 amino acid positions derived from 25 putatively functional genes). All alignments are available from the authors upon request.

### Signature of Selection Analysis

To evaluate the role of natural selection during the evolution of the desaturase gene family in insects, the ratios of nonsynonymous (*d*_N_) and synonymous (*d*_S_) substitution rates were determined using codon substitution models implemented in the software package PAML version 4.4 (Yang 2007). For each First Desaturase subfamily identified in the main phylogenetic analysis (Desat A1 through E), we computed and trimmed an amino acid alignment using MAFFT and Gblocks as described above, and converted it into a codon alignment with PAL2NAL version 14 (Suyama et al. 2006). Maximum-likelihood trees were calculated for each subclade using RAxML based on the LG model as outlined above, and used to inform the PAML analyses alongside the codon alignments. Genes of each subfamily were then analyzed using the following site-, branch-, and branch-site-specific models of codon substitution.

The basic model M0 assumes that the ratio *ω* = *d*_N_/*d*_S_ is invariable among sites and branches (Goldman and Yang 1994). In contrast, the site-specific models M7 and M8 are based on the more realistic assumption that *ω* varies among sites, but not branches (Yang et al. 2000). As M8 allows for a fraction of sites to be under positive selection (*ω* > 1), but M7 does not, this pair forms an LRT of positive selection with degrees of freedom (df) = 2. We also investigated whether selection acted differently on desaturases in ants, *B. mori* and *T. castaneum*, because these taxa are characterized by especially large gene expansions. To this end, we first applied an LRT with df = 1 comparing M0 and a branch-specific model estimating *ω* separately for branches specified a priori (foreground branches, i.e., all branches leading to and within ants, or *B. mori* and *T. castaneum*, respectively) and the background branches. The latter model, which allows for positive selection, was also tested (df = 1) against a null model which fixes *ω* at 1 to determine whether *ω* is significantly higher than 1 along the foreground branches (Yang 1998). Finally, we investigated whether only some sites are under positive selection along the foreground branches by applying the branch-site test of positive selection. This test (df = 1) is based on a branch-site model, which allows *ω* to vary both among sites and branches, and a null model which caps *ω* at 1 (Yang et al. 2005). Substitution rates were not studied in Ifc and Cyt-b5-r genes.

### Gene Expression Analysis

Sex- and caste-specific desaturase gene expression levels were gathered from RNA sequencing (RNA-seq) data of three ant species, *H. saltator*, *C. floridanus* and *P. barbatus*, and RNA-seq data of *H. saltator* and *C. floridanus* were obtained from Bonasio et al. (2010) and are described there in more detail. Briefly, pools of nonreproductive (*H. saltator*) or minor (*C. floridanus*) workers of various ages, queens, and males were used to construct cDNA libraries from poly-A RNA and sequenced on a Illumina 1G Genome Analyzer (Illumina, San Diego, CA) with a paired-end module. Reads were aligned to the most recent genome assemblies using TopHat (Kim et al. 2013), and read counts expressed as RPKM values to account for differences in gene length and total number of reads (Mortazavi et al. 2008).

*Pogonomyrmex barbatus* RNA-seq data were acquired individually from two adult workers and two virgin queens. cDNA libraries were constructed from poly-A RNA using the Nextera XT DNA Sample Preparation Kit (Illumina) and sequenced from both ends on the HiSeq 2000 Sequencing System (Illumina) with current v3 chemistry. Reads were aligned to the *P. barbatus* genome assembly v1.0 using TopHat v2.0.8 (Kim et al. 2013) and mapped to the Official Gene Set v1.2 using Cufflinks v2.0.2 (Trapnell et al. 2010). RPKM values were calculated from read counts as above, and averaged across worker and queen data sets.

Although the *H. saltator* and *C. floridanus* data sets were obtained from specimens raised in the same laboratory and by using the same sequencing protocol, the *P. barbatus* data were acquired under different conditions. In order to control for these differences, we determined gene expression levels (as RPKM values) and variance of seven housekeeping genes commonly used to normalize gene expression across samples (Scharlaken et al. 2008; Cheng et al. 2013). Four of these, including actin and GAPDH, proved to be highly variable within species, which is in line with previous studies advising against their use (reviewed in Bustin 2000). The remaining housekeeping genes, EF1-beta, RPL18, and RPL13A, showed consistency within species (coefficients of variation ≤ 0.38), and to a lower extent, between species (coefficients of variation ≤ 0.70), and were thus used as reference genes allowing the comparison of First Desaturase gene expression levels both within and between species.

## Supplementary Material

Supplementary figures S1–S3 and supplementary tables S1 and S2 are available at *Molecular Biology and Evolution* online (http://www.mbe.oxfordjournals.org/).

Supplementary Data
