# Supplementary material for: Evolution of the Insect Desaturase Gene Family with an Emphasis on Social Hymenoptera
Source: Mol Biol Evol. 2014 Nov 24;32(2):456–71. doi: 10.1093/molbev/msu315 (PMC4298175; doi:10.1093/molbev/msu315)
Supplement: Supplementary Data [file supp_32_2_456__index.html]

Evolution of the Insect Desaturase Gene Family with an Emphasis on Social Hymenoptera — Evolution of the Insect Desaturase Gene Family with an Emphasis on Social Hymenoptera — Supplementary Data 

# Evolution of the Insect Desaturase Gene Family with an Emphasis on Social Hymenoptera

## Supplementary Data

files

**Files in this Data Supplement:**

- Supplementary Data - pdf file
- Supplementary Data - xls file
