# Supplementary material for: Evolution of the Insect Desaturase Gene Family with an Emphasis on Social Hymenoptera
Source: Mol Biol Evol. 2014 Nov 24;32(2):456–71. doi: 10.1093/molbev/msu315 (PMC4298175; doi:10.1093/molbev/msu315)
Supplement: Supplementary Data [file supp_msu315_MBE-14-0523_R1-Desats-SM-Final-141030.pdf]

Supplementary Material

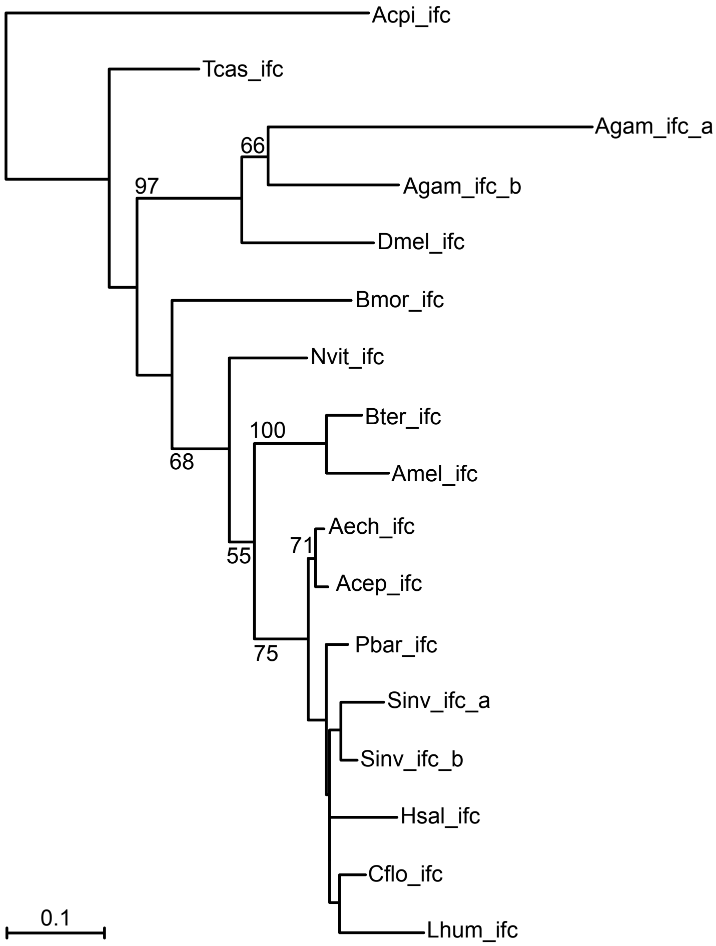

Fig. S1. Reconstruction of the phylogeny of insect Shingolipid Desaturase genes (Ifc subfamily), illustrating the subfamily's single-copy status in 13 of the 15 represented taxa. The unrooted maximum likelihood tree was obtained from 17 genes of 15 species, with confidence values at the edges derived from 1000 rapid bootstrap replicates. Gene names follow the updated nomenclature proposed in this study, based on the previously characterized *ifc* gene of *D. melanogaster*. Species are indicated by four-letter prefixes as follows: Aech = *Acromyrmex echinatior*, Acep = *Atta cephalotes*, Cflo = *Camponotus floridanus*, Hsal = *Harpegnathos saltator*, Lhum = *Linepithema humile*, Pbar = *Pogonomyrmex barbatus* and Sinv = *Solenopsis invicta* (all ants, in color), and Acpi = *Acyrthosiphon pisum*, Amel = *Apis mellifera,* Agam = *Anopheles gambiae*, Bmor = *Bombyx mori*, Bter = *Bombus terrestris*, Dmel = *Drosophila melanogaster*, Nvit = *Nasonia vitripennis* and Tcas = *Tribolium castaneum*.

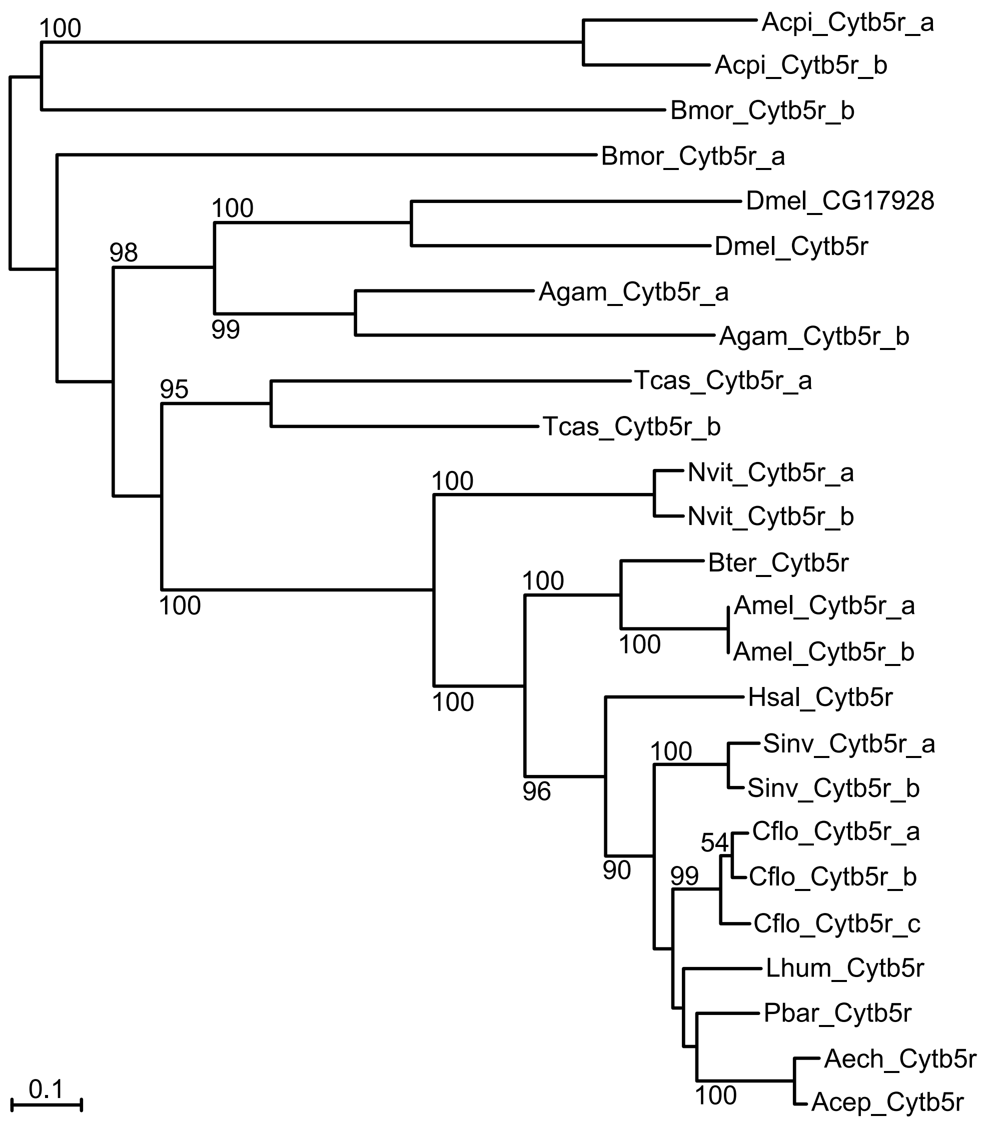

Fig. S2. Reconstruction of the phylogeny of insect cytochrome b5 fused desaturase genes (subfamily Cyt-b5-r), illustrating low numbers of genes (1–3 copies) for all represented taxa. The unrooted maximum likelihood tree was obtained from 25 genes of 15 species, with confidence values at the edges derived from 1000 rapid bootstrap replicates. Gene names follow the updated nomenclature proposed in this study, based on the previously characterized *Cyt-b5-r* gene of *D. melanogaster*. Species are indicated by four-letter prefixes as follows: Aech = *Acromyrmex echinatior*, Acep = *Atta cephalotes*, Cflo = *Camponotus floridanus*, Hsal = *Harpegnathos saltator*, Lhum = *Linepithema humile*, Pbar = *Pogonomyrmex barbatus* and Sinv = *Solenopsis invicta* (all ants, in color), and Acpi = *Acyrthosiphon pisum*, Amel = *Apis mellifera,* Agam = *Anopheles gambiae*, Bmor = *Bombyx mori*, Bter = *Bombus terrestris*, Dmel = *Drosophila melanogaster*, Nvit = *Nasonia vitripennis* and Tcas = *Tribolium castaneum*.

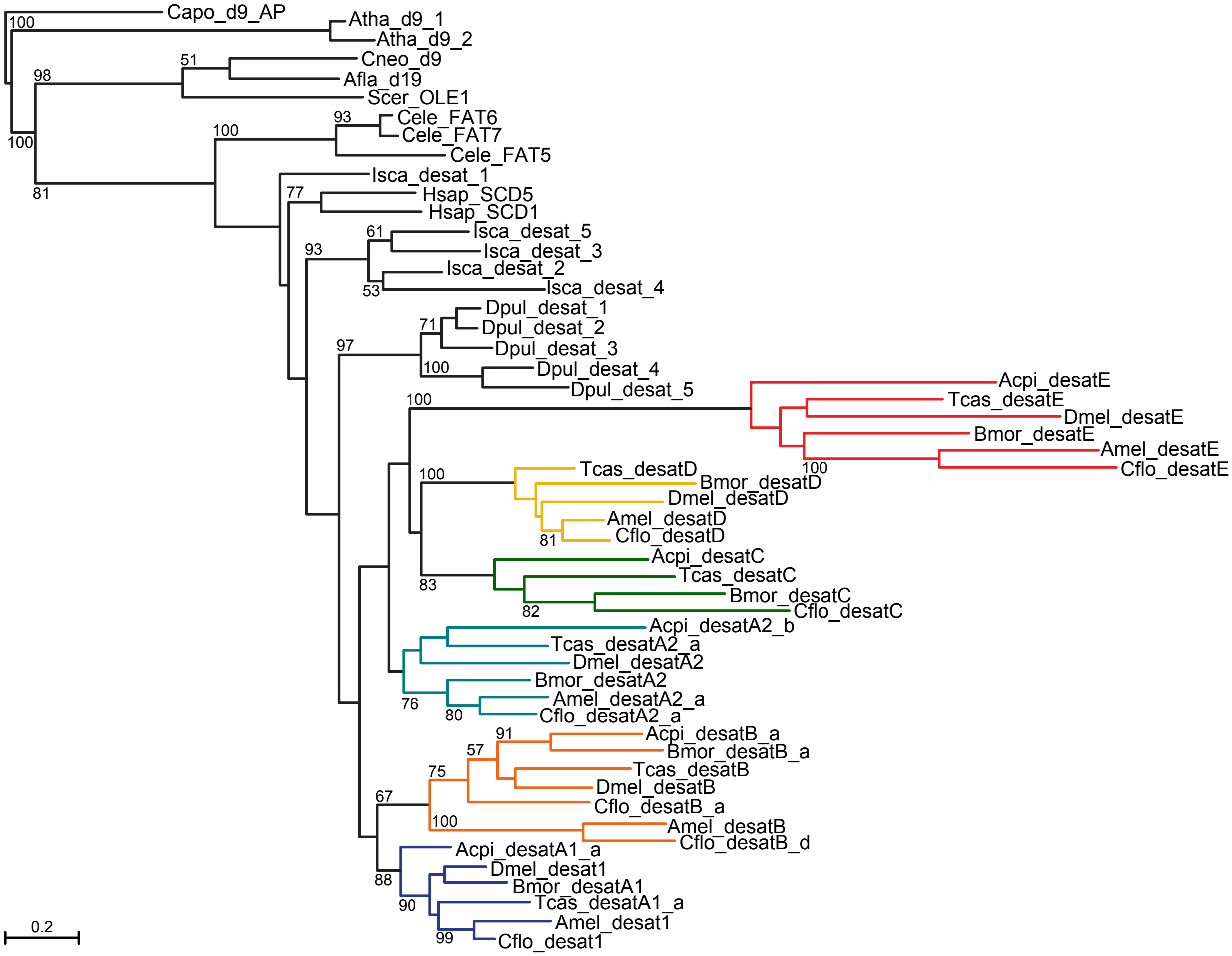

Fig. S3. Phylogenetic reconstruction of eukaryote First Desaturase genes, including representatives of fungi, plants, nematodes, arthropods, and vertebrates. The unrooted maximum likelihood tree was obtained from 55 genes of 15 species, with confidence values at the edges derived from 1000 rapid bootstrap replicates. Species are indicated by four-letter prefixes as follows: Acpi = *Acyrthosiphon pisum*, Afla = *Aspergillus flavus* (fungus, gene XP_002382647.1), Amel = *Apis mellifera*, Atha = *Arabidopsis thaliana* (plant, genes NP_172098.1 and NP_565721.1), Bmor = *Bombyx mori*, Capo = *Cyanobacterium aponinum* (cyanobacterium, outgroup, gene YP_007160219.1), Cele = *Caenorhabditis elegans* (nematode, genes NP_504814.1, NP_001255595.1, and NP_507482.1), Cflo = *Camponotus floridanus*, Cneo = *Cryptococcus neoformans* (fungus, gene XP_567496.1), Dmel = *Drosophila melanogaster*, Dpul = *Daphnia pulex* (water flea, genes EFX60807.1, EFX70200.1, EFX85062.1, EFX82757.1, and EFX82758.1), Hsap = *Homo sapiens* (human, genes NP_005054.3 and NP_001032671.2), Isca = *Ixodes scapularis* (tick, genes XP_002435072.1, XP_002411987.1, XP_002411990.1, XP_002411988.1, and XP_002411989.1), Scer = *Saccharomyces cerevisiae* (fungus, gene NP_011460.3), and Tcas = *Tribolium castaneum*.

Table S1. Genome assemblies and predicted gene sets used to identify and annotate desaturase genes in 15 insect species.

Species	Assembly	Predicted gene set	Source
Acyrthosiphon pisum	v2.0	v2.1b	AphidBase
Tribolium castaneum	v3.0[b]	v3.0	BeetleBase
Bombyx mori	v2.0	GLEAN v1.0	Silkworm Genome Database
Drosophila melanogaster	r5.44	r5.48	FlyBase
Anopheles gambiae	AgamP3	AgamP3.8	VectorBase
Nasonia vitripennis	v2.0[a]	v1.2	Hymenoptera Genome Database
Apis mellifera	v4.5[b]	v3.2	Hymenoptera Genome Database
Bombus terrestris	v1.0	v1.0[a]	Hymenoptera Genome Database
Harpegnathos saltator	v3.3[b]	v3.3	Hymenoptera Genome Database
Camponotus floridanus	v3.3[b]	v3.3	Hymenoptera Genome Database
Linepithema humile	v1.0[b]	v1.2	Hymenoptera Genome Database
Pogonomyrmex barbatus	v1.0[b]	v1.2	Hymenoptera Genome Database
Solenopsis invicta	v1.0	v2.2.3	Hymenoptera Genome Database
Atta cephalotes	v1.0[b]	v1.2	Hymenoptera Genome Database
Acromyrmex echinatior	v2.0[b]	v3.8	Hymenoptera Genome Database

Note— [a] obtained by personal communication and not available publicly as of Apr 28, 2014

[b] in addition, unassembled contigs were available
